# Book Notices

**Published:** 1860-12

**Authors:** 


### BOOK NOTICES.

On Obscure Diseases of the Brain and Disorders of the Mind; Their Incipient Symptoms, Pathology, Diagnosis, Treatment, and Prophylaxis. By Forbes Winslow, M. D. Blanchard and Lea, Philadelphia; 1860. For sale by Wm. Keen, No. 148 Lake Streeet, Chicago, Ill.

The announcement of Forbes Winslow as the author of a work on the subject, is a sufficient guarantee of its excellence; for, as relates to cerebral disorders, he has obtained an undisputed place among the best writers of the present day. An idea of the scope and design of this work may be gathered from the following extract from the preface: "This work was originally intended as an introductory chapter to a treatise I have for some time been preparing for publication, on 'Softening, and other types of Organic Disease of the Brain.' In consequence of the great and unexpected length to which the contemplated prefatory essay extended, it occurred to me that it would be more consistent with a scientific analysis of the subject, to continue my researches, and publish them in a distinct and separate volume, 'On the Incipient Symptoms of Obscure Diseases of the Brain, and Disorders of the Mind,' as *avant courier*, or introduction to the work which is exclusively to relate to the specific types of encephalic disease. Such, briefly, is the origin ot the present treatise." It is safe to affirm, that cerebral diseases, whether physical or mental, are not so readily recognized or well understood as they might be with suitable attention, and the degree of influence that maladies of the mind exert over the body, or on the other hand that diseases of the body have upon the mind are not to their full extent realized, and whoever labors to direct attention to these points, or by his own observations helps to elucidate them, merits the thanks not only of the profession, but of the race. We have neither time nor space for an extended review of the work, and will only add, that any thoughtful physician who will procure and thoroughly read the book, cannot fail to find himself amply rewarded.

An Elementary Treatise on Human Anatomy. By Joseph Leidy, M. D., Professor of Anatomy in the University of Pennsylvania, etc. With 392 Illustrations. J. B. Lippincott & Co., Philadelphia. For sale by S. C. Griggs & Co., 39 and 41 Lake Street, Chicago, Ill.

The author in his preface says: "The present work is intended as an elementary treatise on human anatomy, and is not an elaborate system adapted to the use of those who have already advanced in anatomical knowledge. The author has attempted to prepare such a book as he feels would have been of service to himself in the commencement of his studies, and he hopes it may be found worthy of the approbation of students, for whom, and by whose frequent solicitation, it was written.". The work contains more than six hundred octavo pages, both type and paper being of a superior quality. The illustrations are well selected as to their anatomical interest, and their artistic execution is extremely good. The heads of subjects are set in heavy type so as readily to catch the eye, which renders it a more pleasant work of reference. The anatomical names of parts are accented in such a manner as to enable the student to give them their proper pronunciation, a point upon which even our medical dictionaries are extremely imperfect.

---

The Pocket Anatomist. Being a complete description of the Anatomy of the Human Body. For the use of Students. By M. W. Hilles. Published by Lindsay & Blakiston, Philadelphia, 1860. For sale by S. C. Griggs & Co.

This book, as its name implies, is a condensed compendium of human anatomy, and is designed especially as a work of convenient reference for students while pursuing their anatomical studies, and it seem well adapted to the purpose for which it has been issued.

